# Myocardial fatty acid metabolism probed with hyperpolarized [1-13C]octanoate

**DOI:** 10.1186/1532-429X-17-S1-O101

**Published:** 2015-02-03

**Authors:** Hikari Yoshihara, Jessica A Bastiaansen, Magnus Karlsson, Mathilde H Lerche, Arnaud Comment, Juerg Schwitter

**Affiliations:** 1 Dept. of Cardiology, Lausanne University Hospital (CHUV), Lausanne, Switzerland; 2 Dept. of Radiology, Lausanne University Hospital (CHUV) and University of Lausanne (UNIL), Lausanne, Switzerland; 3 Albeda Research ApS, Copenhagen, Denmark; 4 Institute of Physics of Biological Systems, Swiss Federal Institute of Technology (EPFL), Lausanne, Switzerland

## Background

The heart normally derives most of its energy from the oxidation of fatty acids. Myocardial metabolism can be monitored non-invasively by MRS using hyperpolarized (HP) 13C-labelled compounds; however, the vast majority of studies reported have used HP pyruvate and did not measure fatty acid catabolism. The myocardial metabolism of HP [1-13C]butyrate and [1-13C]acetate has been reported. The conversion of these short-chain fatty acids to acetyl-CoA does not involve successive rounds of beta-oxidation, as is the case for longer chain fatty acids, which are a more important source of cardiac energy. In this study we examined the applicability of hyperpolarized [1-13C]octanoate, a medium-chain fatty acid, as a probe of myocardial metabolism.

## Methods

[1-13C]octanoic acid (4 M in DMSO, doped with stable trityl radical) was polarized by microwave irradiation (196.8 GHz) at 7 T & 1 K. After dissolution with superheated buffered D2O, ~0.04 mmol was infused via a femoral vein catheter into anesthetized Wistar rats in a 9.4 T horizontal bore scanner (Varian) and a series of single pulse (BIR-4, 300, TR ~3 s) gated 13C MRS acquisitions was performed with a surface coil positioned over the heart. To aid metabolite identification, HP [1-13C]acetate and/or 13C-urea were coinfused in several experiments.

## Results

**Figure 1:**
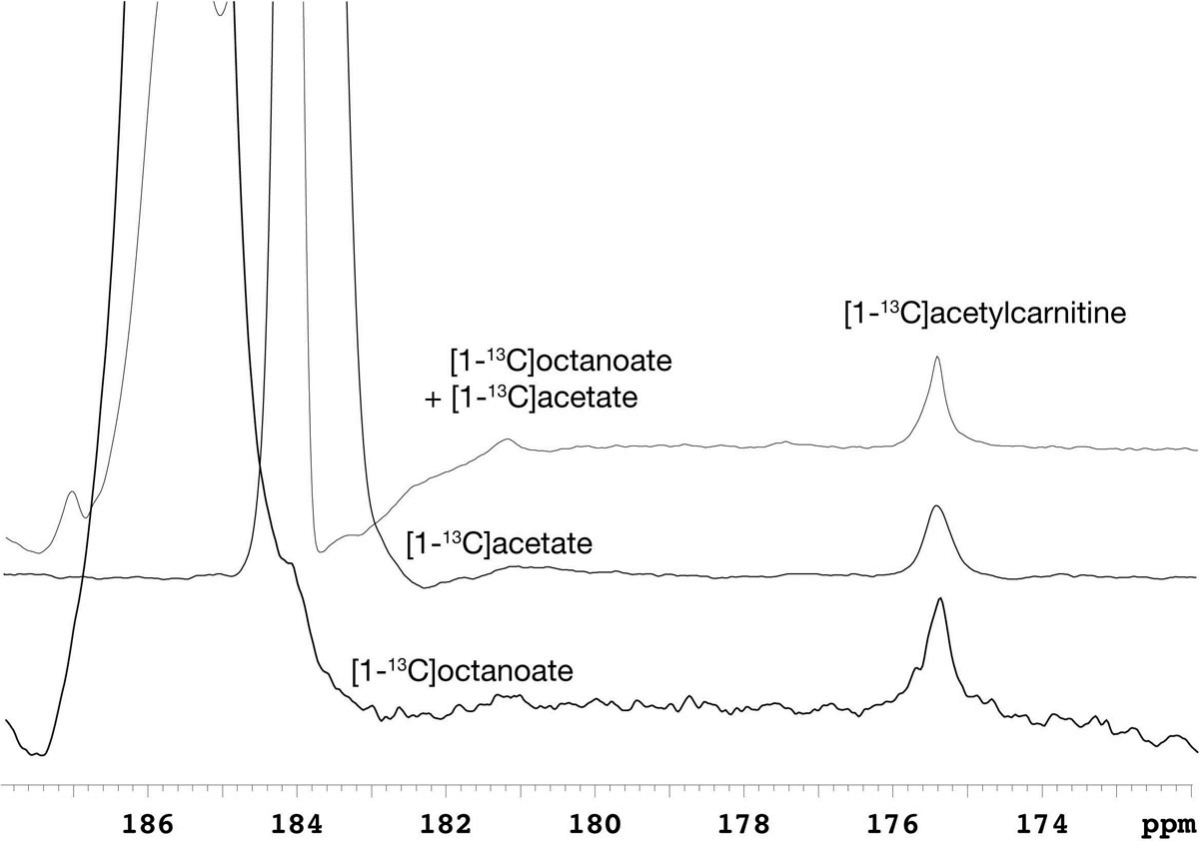
Acetylcarnitine produced in the rat heart from hyperpolarized [1-13C]octanoate. Overlay of summed 13C MR spectra acquired in vivo, chemical shift referenced to coinfused 13C-urea, with hyperpolarized [1-13C]octanoate and [1-13C]acetate infused, separately or together, converted to [1-13C]acetylcarnitine.

After dissolution, [1-13C]octanoate polarization level and T1 relaxation rate were ~11% and 29 ± 3 s, respectively. In vivo, the octanoate signal decayed rapidly and was no longer measurable 20-36 s after the start of infusion. Interactions with blood proteins such as serum albumin are likely responsible for the rapid loss of signal, and the T1 in blood ex vivo was ~9.6 ± 0.5 s. One metabolite peak at 175.4 ppm, was consistently observed (Fig 1). Summing the FIDs where octanoate was present and integrating the peaks, the metabolite had 1.49 ± 0.20% the area of octanoate C-1 (n=5). The chemical shift of the metabolite was assigned to [1-13C]acetylcarnitine. This was confirmed by infusing HP [1-13C]acetate and observing [1-13C]acetylcarnitine with the same chemical shift, as well as coinfusing HP [1-13C]acetate and [1-13C]octanoate and observing a single metabolite peak for [1-13C]acetylcarnitine.

## Conclusions

This study demonstrates that in-vivo dissolution DNP metabolic experiments can be performed with 13C-labelled medium-chain fatty acids. Sufficient 13C polarization in octanoate survives circulation, tissue uptake, mitochondrial transport and conversion by beta-oxidation to acetyl-CoA to be detectible in the acetylcarnitine pool. HP octanoate can be used to directly probe the beta-oxidation of metabolically important fatty acids in the heart.

## Funding

Work supported by the Swiss National Fund (grants #138146 & PPOOP1_133562).

